# An exploratory trial of insulin initiation and titration among patients with type 2 diabetes in the primary care setting with retrospective continuous glucose monitoring as an adjunct: INITIATION study protocol

**DOI:** 10.1186/1471-2296-15-82

**Published:** 2014-05-03

**Authors:** Irene D Blackberry, John S Furler, Louise E Ginnivan, Hanan Derraz, Alicia Jenkins, Neale Cohen, James D Best, Doris Young, Danny Liew, Glenn Ward, Jo-Anne Manski-Nankervis, David N O’Neal

**Affiliations:** 1 General Practice and Primary Health Care Academic Centre, The University of Melbourne, 200 Berkeley St, Carlton 3053, Australia; 2 Baker-IDI Heart and Diabetes Institute, 75 Commercial Rd, Melbourne VIC 3004, Melbourne, Australia; 3 Department of Medicine, St Vincent’s Hospital, The University of Melbourne, Level 4, Clinical Sciences Building, 29 Regent St Fitzroy, Melbourne VIC 3065, Australia; 4 Melbourne Medical School, The University of Melbourne, Level 2 West, Medical Building (181), Melbourne VIC 3010, Australia; 5 Melbourne EpiCentre, The University of Melbourne, c/-The Royal Melbourne Hospital, 7 East, Main Building, Grattan St, Parkville VIC 3050, Australia; 6 Department of Endocrinology and Diabetes, St Vincent’s Hospital, Level 4, Clinical Sciences Building, 29 Regent St Fitzroy, Melbourne VIC 3065, Australia

**Keywords:** Primary care, Family medicine, Insulin, Nurse, Type 2 diabetes, Retrospective continuous glucose monitoring

## Abstract

**Background:**

Insulin initiation and titration in primary care is necessary to respond to the growing epidemic of type 2 diabetes (T2D). The INITIATION study aims to evaluate the impact of implementing a new model of care with Primary Care Physician and Practice Nurse (PN) teams supported by a Credentialed Diabetes Educator-Registered Nurse (CDE-RN) and endocrinologist in initiating and titrating basal and prandial insulin for T2D patients in the Australian healthcare system over 24 weeks. This study also explores the feasibility and efficacy of retrospective continuous glucose monitoring (r-CGM) in comparison with self-monitoring of blood glucose (SMBG) among people with T2D in primary care.

**Methods/Design:**

The study employs a before and after design with a nested exploratory trial of SMBG and r-CGM. A total of 102 insulin naïve T2D patients with a glycated haemoglobin (HbA1c) level of >7.5% in the previous 6 months while treated with maximal oral therapy will be recruited and screened from 22 primary care practices in Melbourne, Australia. All patients will be commenced on a basal insulin regimen following randomization into one of the two blood glucose monitoring arms, with intensification to a “basal plus” regimen if required. The outcomes of the new model of care will be benchmarked with data collected over the same period from a specialist setting in Melbourne, Australia.

**Discussion:**

This article describes the study protocol and insulin treatment algorithm employed in the first study to explore r-CGM use among T2D in primary care. Findings from the INITIATION study will inform development of a larger randomized controlled trial.

**Trial registration:**

ACTRN12610000797077.

## Background

There are currently 347 million people with diabetes world-wide and this number is expected to rise rapidly, particularly those with type 2 diabetes (T2D) [1]. Uncontrolled diabetes results in premature morbidity and mortality and contributes to the burden faced by society and its health systems [1]. Optimal glycemic control among people with T2D is essential to reduce the risk of developing macro and microvascular complications [2]. Yet, despite available evidence based guidelines for diabetes management, there is a persistent failure to achieve glycemic targets among almost half of those people diagnosed with T2D [3,4].

The role of insulin in the management of T2D is well established [5]. However, insulin is often initiated too late in the disease progression of T2D. Data from the INSTIGATE study conducted in Europe showed that the average glycated haemoglobin (HbA1c) upon which insulin was initiated was 9.2% [4]. In Australia, the Fremantle Diabetes Study, reported a median HbA1c of 9.4% prior to insulin commencement [6]. Even when insulin is initiated as part of T2D management, the initial insulin regimen is often not intensified, leaving the average HbA1c still well out of target, with only 17% of people commenced on insulin achieving target HbA1c [7,8]. Given the growing prevalence of T2D and the limited availability of diabetes specialist resources, insulin initiation and titration in primary care is necessary for uncomplicated patients [9,10].

A single daily dose of basal insulin is used in nearly two thirds of primary care patients with T2D treated with insulin [4,11]. However the majority of people with T2D and elevated fasting glucose levels also have post-prandial hyperglycemia [12]. Over two-thirds of patients in the 4-T study needed more than one type of insulin to achieve target HbA1c suggesting that once insulin is initiated, monitoring and intensification of insulin therapy is required [13]. The “basal plus” insulin regimen addresses the meal with the highest post-prandial glucose levels after fasting glucose levels are at target following the introduction and up-titration of basal insulin [14].

Assessing post-prandial glycemia is regarded as the most significant obstacle in the initiation and titration of rapid-onset, short-acting prandial insulin. Obtaining high quality post-prandial readings from patients is often a challenge [15]. Retrospective-continuous glucose monitoring (r-CGM) devices provide more reliable and detailed glucose recordings post meals. Use of these devices thus may increase the timeliness and safety of insulin initiation and up-titration among people with T2D, particularly by identifying asymptomatic nocturnal hypoglycemia and unrecognized post-prandial glucose elevations [16,17]. This technology has been used widely in type 1 diabetes (T1D) in specialist centers, however evidence regarding the utility and efficacy of this technology in primary care is lacking [18-20]. While attention needs to be paid to the technical evidence relating to insulin introduction, addressing barriers to the initiation of insulin and tailoring the interventions at the levels of patient, health professional and health system will ensure the interventions are widely acceptable and sustainable in primary care [21-23].

The primary aim of this study is to evaluate the feasibility and glycemic outcomes resulting from the implementation of a new model of care in primary care [24] facilitating the addition of a basal +/- prandial insulin regimen in T2D patients with inadequate glycemic control on oral therapy alone. It involves primary care physician/Practice Nurse (PN) teams with support in a “hub-and-spoke” fashion from Credentialed Diabetes Educator – Registered Nurse (CDE-RN) and endocrinologists. The secondary aim of the study is to evaluate the performance and clinical impact of r-CGM in guiding insulin dosing in primary care.

## Methods/Design

**Figure 1:**
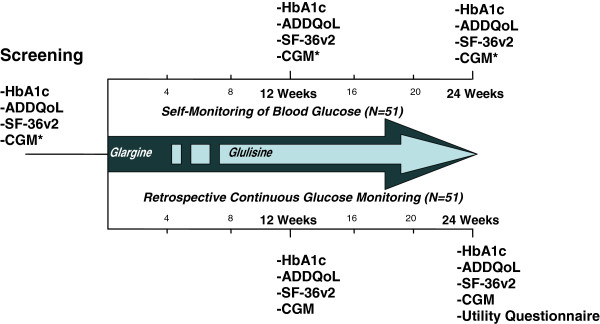
Overview of the INITIATION study protocol.

The INITIATION study incorporates a model of care with the PN and primary care physician working as a team to initiate and titrate basal and prandial insulin in the Australian primary care setting. The new model of care is evaluated using a before and after study design. Resources have been developed to guide these primary care teams to effectively implement the study protocol (Figure 1). Support and mentorship will be provided to primary care physicians and PNs by the study CDE-RN and the study endocrinologist. Nested within this study is an exploratory trial of two different types of blood glucose monitoring. Participating T2D patients will be randomized to one of two arms, namely the self-monitoring of blood glucose (SMBG) alone (Freestyle Optium™; Abbott) or with an adjunct r-CGM (*i*Pro™2/Enlite™; Medtronic). Each patient will be followed for 24 weeks post-randomization with outcomes related to measures of glycemia (identification of elevated post-prandial glucose levels and the time required to reach target glycemia), quality of life and utilization of health professionals’ time spent with patients.

### Ethics approval and trial registry

The INITIATION study has been approved by St Vincent’s Health Human Research Ethics Committee (HREC D-106/10) and was registered with the Human Research Ethics Committee at the University of Melbourne (HREC 1135260) and the Baker-IDI. Consent will be obtained from participating primary care team and patients. The INITIATION study was registered with the Australian and New Zealand Clinical Trials Registry (ACTRN12610000797077).

### The intervention

Strategies and materials in the INITIATION study, including the model of care, recruitment and study protocol, are adapted from an acceptability and feasibility study in five primary care practices in metropolitan Melbourne [24].

### Training in the model of care

All participating primary care physicians and PNs will receive a 2-hour education and training session led by the study endocrinologist, primary care physician and/or CDE-RN. The focus of the training will be on establishing a *collaborative model of care* which includes:

• The primary care physician-PN team working in partnership to identify and support the initiation of insulin in appropriate patients.

• An in-practice system to initiate and titrate insulin (e.g. detailed description of the roles of the team members and within-practice systems of referral and communication between primary care physician and PN).

• Simple protocols and insulin titration algorithms.

• Study endocrinologist and CDE-RN support as appropriate and necessary.

**Figure 2:**
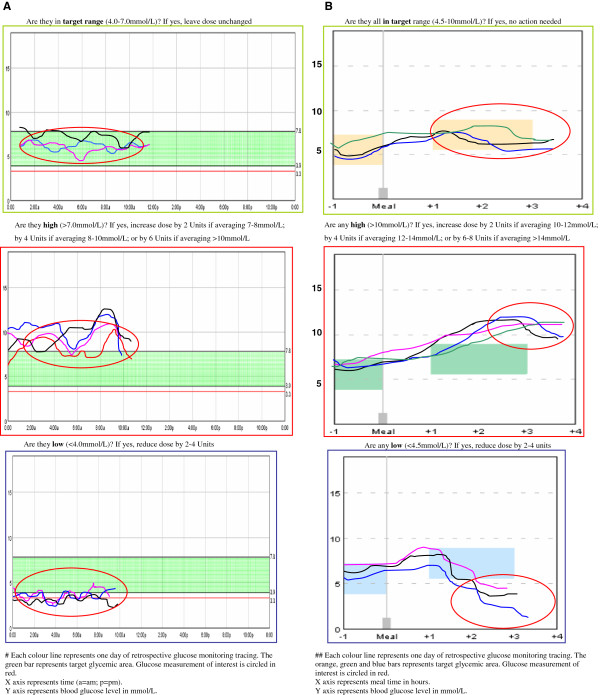
**Glargine and glulisine titration schedule based on r-CGM traces. A** Glargine titration schedule based on r-CGM traces using the average glucose levels overnight^#^. **B**. Glulisine titration schedule based on r-CGM traces using the glucose levels following meals^##^.

The training of the primary care physician and PN teams will be interactive and hands-on, using case studies and delivered either in-practice or during an evening group session. The session will cover evidence and rationale for insulin use, how to motivate patients and deal with common patient-level barriers to insulin initiation, initiating and titrating basal insulin (glargine; Sanofi) and prandial insulin (glulisine; Sanofi) using a structured protocol (Table 1 and Figure 2A and 2B). The same type of disposable injecting device (Solostar™, Sanofi) will be used for both insulin glargine and glulisine. The training will involve demonstration of these insulin devices and the r-CGM device (*i*Pro™2/Enlite™; Medtronic).

**Table 1:** Insulin titration algorithm

Fasting BGL (mmol/L)*	**Glargine titration**	2 hr post prandial BGL (mmol/L)*	**Glulisine titration**
<4.0	Reduce by 2–4 units	<4.5	Reduce by 2–4 units
4.0-7.0	No change	4.5-7.0	No change
7.1-8.0	Add 2 units	7.1-10.0	Add 0–2 units^#^
8.1-10.0	Add 4 units	10.1-12.0	Add 2 units
>10.0	Add 6 units	12.1-14.0	Add 4–6 units
		> 14.0	Add 6–8 units^#^

BGL = Blood Glucose Level.

#Upon GP’s discretion.

*For values in mg/dl multiply by 18.

The aim of the training is to ensure that the primary care physician/PN teams have the knowledge, systems, supports and confidence to work collaboratively with participating patients to commence insulin. Importantly, referral or consultation with an endocrinologist, CDE-RN or any other appropriate health professionals will remain as management options for primary care physicians managing study patients.

### Intervention with participating patients

A scheduled visits manual has been developed to guide primary care physicians and PNs to follow-up participating patients allowing clinical flexibility within general practices to implement the study protocol in a real-world primary care setting.

The baseline assessments and enrollment visits with the primary care physician and PN will be arranged for each participating patient by practice staff. At this visit the PN will lead the discussion with the patient around general diabetes management and education and train patients to administer a single injection of insulin glargine with the Solostar™ injecting device. PNs will then review patients on Day 2 using a checklist and will supervise patients administering the second injection of insulin glargine themselves. While patients will be advised to administer glargine at the same time daily, they can choose the most appropriate time that will fit in with their daily activities.

**Figure 3:**
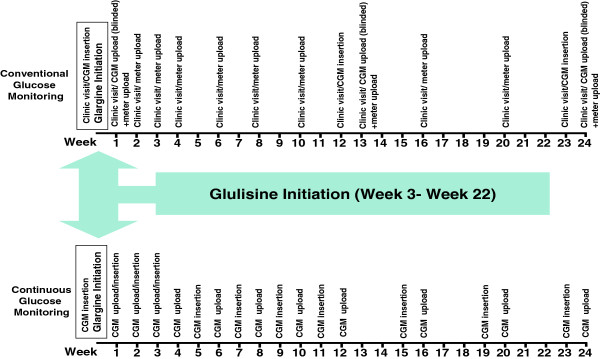
Overview of monitoring schedule.

The primary care teams will be provided with a simple dosing protocol (see Table 1 and Figure 2) to initiate and titrate insulin with options to deviate from this protocol according to their clinical judgment. The protocol is to target fasting glucose level with once daily insulin glargine starting from 10 units, although this can be modified at the discretion of the physicians. Oral hypoglycemic agents will remain unchanged at the time of basal insulin initiation in order to ensure that glycemia will not deteriorate further prior to up-titration of the insulin in a group of patients with glycemic control that is already suboptimal. The subsequent titration visits will occur according to a structured schedule (Figure 3). An assessment for the need for prandial insulin can occur at any time four weeks post initiation of basal insulin. After the fasting glucose level target is reached, the primary care teams will be instructed to target the highest post-prandial rise with a pre-meal insulin glulisine injection starting from 4 units with titration guidelines provided (Table 1). Again the subsequent titration visits will occur according to a structured schedule (Figure 3).

The initial dose and titration schedule for insulin glargine (Table 1) is modified from the AT.LANTUS study [25]. The initial dose of insulin glulisine is based on the INITIATE study while the titration schedule (Table 1) is adapted from the study by Janka et al. [26,27]. Titration algorithms for the two monitoring arms (SMBG and r-CGM) are designed so as to provide equivalent increments in insulin in response to measured glucose readings. They are also designed to minimize the risk of hypoglycemia. The study CDE-RNs and endocrinologist will continue to provide support and mentoring to primary care physicians and PNs during insulin initiation and titration as required and will perform safety monitoring by regular review of glucose uploads.

### Study procedures

#### ***Inclusion criteria and recruitment strategies for primary care practices***

Inclusion criteria for primary care practices include:

• The primary care practice employs a PN;

• Primary care physicians and PNs have not previously routinely initiated insulin in their practice.

A list of potential practices in the state of Victoria, Australia will be identified from the primary care practice database at the University of Melbourne and referral network of the study investigators (St Vincent’s Hospital, Werribee Mercy Hospital and Baker-IDI Heart and Diabetes Institute). Recruitment will commence within metropolitan Melbourne. An invitation letter and a study flyer will be mailed to eligible practices. Primary care physicians from eligible practices will be contacted by one of the study investigators. Practices who express an interest in participating will receive an in-practice briefing visit by one of the study team. The purpose of the briefing visit is to explain the study in more detail and to obtain written consent from the primary care physicians and PNs to participate in the study.

#### ***Inclusion criteria and recruitment strategies for patients***

Each consented practice will undertake an audit to identify potential patients with the most recent HbA1c > 7.5% in the last 6 months from their electronic medical records and/or pathology providers. Patients who meet the inclusion and exclusion criteria will be sent a letter by their primary care physicians recalling them for review of their diabetes control. During this review, the need for insulin as one of the treatment options available will be discussed with the patient. Patients will be provided with written study information and will be asked to attend a screening visit with the PN to confirm eligibility to participate.

Each potential patient will be screened by the PN according to the study inclusion criteria:

• Uncomplicated, insulin naïve T2D patients.

• Aged 18–80 years old.

• On maximum tolerated doses which have been stable for at least 3 months of two or more oral hypoglycemic agents (OHAs) or in the opinion of the physician insulin is deemed necessary.

• Willing to self-monitor blood glucose levels at least twice daily.

• Willing to commence insulin.

The study exclusion criteria are:

• Patients with T1D.

• Having satisfactory fasting glucose levels (mean <6.0 mmol/L) with post-prandial hyperglycemia (mean >10 mmol/L).

• Having previous or current treatment with insulin (excluding the short term use of insulin in acute illness or during hospitalization).

• On Glucagon-like peptide-1(GLP-1) medication

• Non-English speaking.

• Having vision/cognition/renal function impairment or having major medical/psychiatric illness.

• Having recent or ongoing life-threatening illness.

• Having needle phobia.

• Pregnant or planning to be pregnant.

#### ***Screening and baseline assessment***

At the screening visit and after written consent is obtained, patients will be provided with and will be taught to use the study blood glucose meter which will be provided as well as general diabetes education including foods containing carbohydrates, the importance of regular exercise, brief explanation of progression of T2D and why insulin is now needed, and a blood glucose monitoring record book. Patients will be asked to perform twice daily glucose levels prior to the next appointment. An r-CGM device will be attached to all patients during this visit allowing collection of a week’s r-CGM trace prior to randomization. Patients will then have blood drawn for HbA1c at the local Diabetes Control and Complications Trial (DCCT) aligned pathology provider designated for the study.

Within a week of screening, patients will visit their primary care team for a baseline assessment and enrollment visit. Those with a study HbA1c ≤7.5% will be excluded from the study and will be referred back to usual care. Patients who meet the inclusion and exclusion criteria will be enrolled into the study and will be asked to complete baseline surveys prior to randomization to either self monitoring of blood glucose (SMBG) alone (Freestyle Optium™; Abbott) or with adjunct r-CGM (*i*Pro™2/Enlite™; Medtronic). Randomization will be generated using computerized random number table that will be held by a researcher independent of the study team. Random allocation will be kept in sealed envelopes and randomization will occur prior to the initiation of insulin therapy. Blinding of researchers, practices and patients will not be possible once the random allocation is revealed due to the nature of the study.

#### ***Self-monitoring arms***

• *Self-monitoring of blood glucose with adjunct (r-CGM) arm*

• *i*Pro™2/Enlite™; Medtronic is a r-CGM utilising a subcutaneous electrode measuring and recording interstitial glucose levels using glucose oxidaze methodology and requiring twice daily calibration with a fingerprick glucose meter reading. Minimal interaction by the patient is required with these devices which are uploaded to a computer. Calibrating fingerprick glucose readings are automatically integrated and an r-CGM trace facilitated by the device software plotted. Health professionals will upload data from the r-CGM device and the blood glucose meter (BGM) at the end of monitoring period and the retrospective continuous glucose trace plot will be produced by the device software. The 7-day r-CGM traces will be collected weekly for 4 weeks following the initiation of insulin glargine or glulisine, fortnightly for another 4 weeks and monthly until the end of the study.

• *Self-monitoring of blood glucose (SMBG) arm*

• For patients randomized to the conventional SMBG arm, r-CGM traces will be obtained at baseline, 12 weeks and 24 weeks. Health professionals will be instructed not to use upload of these r-CGM traces for titration.

#### ***Data collection***

At baseline, demographic data, clinical history (year of diagnosis, complications, medications, frequency of self-monitoring, hospitalization and hypoglycemia in the past 3 months), smoking status, clinical examination (body mass index, waist-to-hip ratio, blood pressure), Short Form 36 Health Survey questionnaire version 2 (SF-36 v2) [28] and Audit of Diabetes Dependent Quality of Life (ADDQoL) [29] will be collected. The same data will also be collected at 12 weeks and 24 weeks. The doses of basal and prandial insulin administered during study follow-up will be recorded. Data will be collected in clinical record forms (CRFs) that are designed to be user-friendly and easy to administer by busy health professionals working in a complex clinical environment. The CRFs will be faxed from primary care centers to a central electronic database management service.

Each patient will be asked to perform fingerprick capillary blood glucose testing 2 to 4 times daily including one fasting reading before breakfast and another reading at 2 hours after a meal at various times. Patients will be advised to document these readings and any symptomatic hypoglycemic episodes in the blood glucose diary provided. The glucose meter data will be uploaded at each practice visit.

#### ***Blood collection and assays***

HbA1c will be collected at baseline, 12 weeks and 24 weeks and analyzed by a DCCT aligned centralized laboratory.

#### ***Follow-up and endpoint determination procedures***

The primary endpoint is the absolute change in HbA1c following insulin initiation at baseline compared with 24 weeks. The secondary endpoints include changes r-CGM time spent in low (<4.0 mmol/L), target (4.0-10.0 mmol/L) and high (>10.0 mmol/L) glucose range at baseline, 12 and 24 weeks; symptomatic hypoglycemia; incidence of severe hypoglycemia (hypoglycemia resulting in loss of consciousness or requiring third party assistance); proportion of patients being prescribed a prandial insulin (glulisine) injection; time to reach target HbA1c ≤7%; proportion of patients achieving an HbA1c ≤7% at 12 and 24 weeks); total insulin dose at 12 weeks and 24 weeks; change in quality of life (ADDQOL and SF-36v2 at 12 weeks and 24 weeks compared with baseline); blood pressure (12 weeks and 24 weeks compared with baseline) and weight change (BMI and waist-to-hip ratio at 12 weeks and 24 weeks compared with baseline).

Endpoints for the sub-study comparing r-CGM with SMBG reflect those described above. In addition, patients and PNs’ acceptability and utility of CGM devices at 24 weeks and total primary health care utilization at 24 weeks will be examined.

#### ***Benchmark data***

The glycemic outcomes reflected by changes in HbA1c following implementation of this new model of care in primary care setting will be benchmarked against observational data collected from all patients with T2D commenced on insulin by private endocrinologists/CDE-RN teams over the same period. Data to be collected from benchmark patients at baseline, 12 weeks and 24 weeks include age, duration of diabetes, the presence of diabetic complications (laser requiring retinopathy, ischemic heart disease, lower limb ulceration or amputation, stroke), the frequency of home blood glucose monitoring, the need for an interpreter, employment status, whether they live alone, hospitalization within the last 12 weeks, current medications and HbA1c. These characteristics will allow a comparison of the demographic, costs and outcomes of those patients with T2D commencing insulin in the specialist centers with those in primary care.

#### ***Sample size calculation***

The power calculation was based on one sample comparison of mean against a reference value. The HbA1c reference value was 8.23% as reported in the INSTIGATE study [4] assuming no change in HbA1c without the intervention. The new model of care was estimated to result in HbA1c reduction of 0.4%. Based on a standard deviation of 1.44 in the FIELD study [30] with a two-sided alpha of 0.05 and 80% power, a sample size of 102 patients is required to detect change in the primary outcome. Our previous research suggests that in an average two full-time-equivalent primary care physician practice we expect to identify 80–100 patients with T2D. Of these we anticipate 10% (8–10) will meet our inclusion criteria and 50% recruitment rate, generating on average 4–5 participating patients per practice. A total of 22 primary care practices will be required. Our sample is not powered to detect change between SMBG and r-CGM but rather to explore the feasibility and efficacy of r-CGM in primary care.

#### ***Statistical analysis***

The primary analysis will be the change in HbA1c based on the entire sample at baseline, 12 and 24 weeks. This analysis will produce a conservative estimate of the benefit of insulin therapy as glycemic control tends to rise without the introduction of insulin in this population. The changes in HbA1c will be benchmarked against those obtained from patients under specialist care who have been initiated on insulin. A secondary analysis will examine the absolute change in HbA1c between the r-CGM and SMBG groups. Comparisons of the entire sample as well as between r-CGM and SMBG will be made at baseline, 12 and 24 weeks for all secondary endpoints.

#### ***Trial monitoring***

A safety monitoring committee consisting of study endocrinologist, primary care physician and CDE-RNs will be appointed. All significant adverse events (which include hospital presentations or admissions) and their duration will be documented. All serious adverse events will be reported to the safety monitoring committee within 24 hours of the primary care team being made aware of them. Each adverse event will be independently reviewed by other chief investigators (consisting of 4 endocrinologists) independent of the safety monitoring team. Additionally, the Human Research Ethics Committee will be notified of all significant adverse events in writing. Criteria for withdrawal of study participants include major protocol violation, major adverse event directly related to the intervention, pregnancy, voluntary withdrawal of consent or development of life-threatening illness.

## Discussion

Delays in the timely initiation of insulin are often attributed to “psychological insulin resistance” [31] on the part of patients and “clinical inertia” on the part of health professionals. However, barriers associated with the health system responsible for the provision of medical services are also important. The model of care under evaluation in this study attempts to address barriers which may be responsible for delaying insulin initiation in primary care while also utilizing those strengths inherent in the primary care model. Our previous work [21] has identified that the organization within the practice, clarification of the roles and expectations of patient and health professionals involved in insulin initiation and delegation of responsibility for the provision of care are key factors in determining success. Our study introduces a new model of care in Australia, with an enhanced role for the PNs working in partnership with the primary care physicians, supported by CDE-RNs and endocrinologists. This model addresses the barriers and enablers of insulin initiation in the primary care setting at the practice, health professional and patient level. It also utilizes the current healthcare system and financial structures in Australia ensuring immediate generalizability.

To maximize acceptance in a busy and varied clinical environment, the tools designed to implement the intervention are flexible with a facility to be tailored to each practice’s structure and the individual needs of patients. The insulin initiation and titration protocol employs a simple unambiguous easy-to-follow algorithm guiding primary care physicians and PNs in a busy general practice setting. A basal insulin analogue is employed as it would minimize the risk of hypoglycemia. This precaution is crucial in order to ensure that the physician’s, PN’s and patient’s trust in the ability of the model of care to deliver a positive outcome is maintained. Insulin glargine is chosen because it is the only available basal insulin that is subsidized by the Pharmaceutical Benefits Scheme (PBS) for the management of T2D in Australia at the time this study is conducted. A basal plus approach, incorporating the rapid acting analogue, insulin glulisine, acknowledges the importance of addressing post-prandial hyperglycemia in reducing HbA1c in this patient group [32,33]. A similar disposable injecting device (Solostar™, Sanofi) mechanism used for both glargine and glulisine minimizes need for further education of primary care physicians, PNs and patients.

The insulin titration schedule is conservative, balancing sophistication against practicability. Its aim is to minimize hypoglycemia risk, and has been developed based upon the review of available research evidence [14] and clinical experience. Each physician-PN team will be able to deviate from the insulin initiation and titration protocol at their discretion. This should minimize patient adverse events and ensure that it is feasible for both PNs and physicians to implement this model of care within their current workload in real-world practice. Our aim is to embed and integrate the task of initiating and titrating insulin within the complex ongoing generalist work of primary care with minimal disruption to work in general.

### The use of blood glucose monitoring among T2D in primary care

Once insulin has been initiated, the ongoing monitoring and review of glucose levels by the patient and health professional is important for patient safety and for intensification of insulin therapy [13] as this provides insight into recurring patterns of glycemia, the impact of diet and behavior upon glycemia and the effect of therapeutic interventions [34]. However hypoglycemia may be undetected particularly when it occurs overnight [35] and identifying post-prandial glycemia may be a challenge for clinicians.

The use of r-CGM offers the potential for identification of unrecognized hypoglycemia and hyperglycemia excursions [36] leading to more tailored and appropriate treatment changes. We chose the *i*Pro™2 r-CGM device as it is small, convenient and non-intrusive for the patient to wear and is essentially “wear and forget”. It requires a minimum of two blood glucose meter readings each day for calibration. The study protocol will result in the first widespread use of these devices in a “real world” primary care setting where people with T2D receive the majority of their health care. Given the exploratory nature of the r-CGM arm of the study the design incorporated an intensive r-CGM regimen providing a level of redundancy sufficient to allow for potential device failures.

### Study design issues

We have undertaken a series of studies of insulin initiation in primary care based on the UK Medical Research Council (MRC) complex intervention framework [37]. In the pre-clinical phase, we examined barriers and enablers of insulin initiation using qualitative approach [21] drawing upon the Normalization Process Theory [38]. We then developed and piloted our new model of care on insulin initiation in five general practices in Melbourne, Australia as part of Phase 1 modeling stage of the MRC framework [39]. Our new model of care is complex as it is made of several interconnecting factors [40] and it is embedded within the ‘real world’ general practice setting. In the INITIATION study, we will progress to a Phase 2 exploratory trial to further refine our intervention and processes as well as to determine the feasibility of our study protocol in a much larger sample of 22 general practices. The exploratory trial stage is considered important to inform a future definitive randomized controlled trial.

Our study is based in primary care involving “research naive” practices. Data collection forms and titration algorithms need to be easily understood and not time consuming to complete and follow as the practices will receive limited reimbursement for their participation in research. The primary aim of implementing the new model of care is not only to achieve insulin initiation but also the treatment intensification required to achieve appropriate glycemic control in a safe manner. The number of people who transition to prandial insulin and timeliness with which that occurs is therefore also of importance and this is reflected in the study’s secondary endpoints. Other secondary endpoints include indicators clinically relevant to health professionals and patients when commencing insulin, including quality of life and weight gain.

Our primary care practices will be a convenience sample of practices in the state of Victoria and thus will not be representative of all Australian primary care practices. A convenience sample is within the scope of this before and after study with a nested exploratory trial to examine the impact of our new model of care and observe the feasibility of r-CGM use among T2D in primary care. Data relating to insulin initiation from a specialist setting in the same locality as that from which the practices will be recruited will be used to benchmark study outcomes. This is incorporated into the study design as assigning patients with suboptimal glycemic control judged by their primary care physicians as requiring insulin on clinical grounds to a control “no insulin” group is not ethically justifiable. Insulin initiation and titration in the specialist setting is accepted as the “gold standard” care and the benchmarking data will enable us to compare how our new primary care based model of care performed in improving HbA1c at 12 and 24 weeks. The benchmarking data will be the basis for a health economics analysis comparing the costs of insulin initiation in the primary care setting versus that in the specialists setting. A centralised laboratory for HbA1c testing will be used in this study to ensure that our study HbA1c result is consistent across participating primary care practices.

Cluster randomization of monitoring arms according to the primary care centres recruited for the study would have been appropriate had we planned a definitive effectiveness trial powered to identify difference in the change in HbA1c. Given that the study is not powered to do so, our individually randomized exploratory design will allow all practitioners to gain some experience and exposure to the use of r-CGM, thus maximizing our capacity to identify strengths and weaknesses in its use in the field.

## Conclusions

Insulin initiation in primary care is warranted in order to address the growing epidemic of T2D. The INITIATION study evaluates the efficacy of a new model of primary care physicians and PNs care that addresses barriers to insulin initiation within real-world primary care setting. The study will be the first to provide valuable efficacy and utility data on the use of r-CGM among people with T2D in primary care.

## Competing interests

We wish to declare the following facts which may be considered as potential conflicts of interest and to significant financial contributions to this work:

(1) Sanofi and Medtronic provided material and financial support for the conduct of this investigator initiated study;

(2) Abbott and BD provided material support;

(3) IDB and JF received fellowship support from NHMRC Centre of Clinical Research Excellence in Diabetes;

(4) JF was also supported by an NHMRC-PHCRED Career Development Fellowship;

(3) LG and HD received travel support to attend a national conference;

(4) NC, DL, AJ, JF, LG and JMN had various financial relationships with pharmaceutical industries *outside the submitted work* including consultancies, grants, lectures, educational activities and travel;

(5) All the other authors had no conflict of interests that may be relevant to the work under consideration.

## Authors’ contributions

All the authors have substantial contributions towards the conception and design of the study. IDB, JF, DY, and JB conceived and developed the insulin initiation model of care. DNO, IDB, JF and AJ conceptualised the Initiation study. DY, JB, LG, NC, DL, HD, JMN and GW contributed to refinement of study protocol, trial design, insulin algorithm, outcomes, and data collection tools. IDB was the study coordinator and wrote the first draft of the manuscript. All authors contributed to subsequent drafts and approved the final version of the manuscript. DNO is the principal investigator and acts as guarantor for the study.

## Pre-publication history

The pre-publication history for this paper can be accessed here:

http://www.biomedcentral.com/1471-2296/15/82/prepub
